# Effects of Mycotoxins on Mucosal Microbial Infection and Related Pathogenesis

**DOI:** 10.3390/toxins7114484

**Published:** 2015-10-30

**Authors:** Seong-Hwan Park, Dongwook Kim, Juil Kim, Yuseok Moon

**Affiliations:** 1 Laboratory of Mucosal Exposome and Biomodulation, Department of Biomedical Sciences, Pusan National University School of Medicine, Yangsan 50612, Korea; E-Mails: dfjjang@naver.com (S.-H.P.); 1022myths@hanmail.net (J.K.); 2 Research Institute for Basic Sciences and Medical Research Institute, Pusan National University, Busan 46241, Korea; 3 National Institute of Animal Science, RDA, Wanju 55365, Korea; E-Mail: poultry98@korea.kr; 4 Immunoregulatory Therapeutics Group in Brain Busan 21 Project, Busan 46241, Korea

**Keywords:** Mycotoxins, microbial infection, mucosal pathogenesis

## Abstract

Mycotoxins are fungal secondary metabolites detected in many agricultural commodities and water-damaged indoor environments. Susceptibility to mucosal infectious diseases is closely associated with immune dysfunction caused by mycotoxin exposure in humans and other animals. Many mycotoxins suppress immune function by decreasing the proliferation of activated lymphocytes, impairing phagocytic function of macrophages, and suppressing cytokine production, but some induce hypersensitive responses in different dose regimes. The present review describes various mycotoxin responses to infectious pathogens that trigger mucosa-associated diseases in the gastrointestinal and respiratory tracts of humans and other animals. In particular, it focuses on the effects of mycotoxin exposure on invasion, pathogen clearance, the production of cytokines and immunoglobulins, and the prognostic implications of interactions between infectious pathogens and mycotoxin exposure.

## 1. Introduction

Mycotoxins are natural, low-molecular-weight secondary fungal metabolites that are detected in various agricultural commodities and humid indoor environments, such as water-damaged buildings [1,2,3,4]. Exposure to mycotoxins in human and animals affects the host immune responses to infectious agents. Various immune-related organs or tissues are impaired by mycotoxins, which alters the susceptibility to the pathogens. Moreover, mycotoxins themselves alter the virulence of the infectious pathogens, leading to changes in the toxicity and invasiveness of the microbes in diverse organs or immune cells [5,6]. The most well-known crosstalk between mycotoxicosis and infection is the aflatoxin B1 exposure with the chronic hepatitis B virus infection, which has synergistic effects on the risk of hepatocellular carcinoma [7,8]. The main sites of mycotoxin exposure are the mucosal epithelia in the gut and airways with the underlying mucosal lymphoid tissues. In particular, the present review examines the interaction between mycotoxins and mucosa-associated pathogenic bacteria or viruses; this interaction exerts detrimental effects on target organs or cells by altering physiological or immunological conditions. The purpose of this review is to provide insights into the crucial roles of mycotoxins (including deoxynivalenol, fumonisin, T-2 toxin, aflatoxin, and ochratoxin) in the pathogenesis of mucosa-associated pathogens and their related mucosal disorders.

## 2. Mycotoxin Exposure and Altered Host Immune Responses

Various mycotoxins affect immune-related organs and cells, and influence host defenses against infectious agents and related microbial toxins [9]. Many previous studies have shown that aflatoxins suppress immune functions, particularly cell-mediated immune responses [10,11]. For instance, high levels of aflatoxin B_1_ (AFB_1_)-albumin adducts change T-cell phenotypes and reduce the percentage of B cells in human immunodeficiency virus-positive individuals [12]. Moreover, exposure to ochratoxin A (OTA) results in reductions in the sizes of crucial immune organs in various species, including the thymus [13] and bursa of Fabricius with advanced atrophy of bursal follicles [14,15,16]. OTA induces necrosis of the germinal center of the spleen and lymph nodes in rats and dogs. Mice exposed to OTA have a decreased number of hematopoietic stem cells [13] and total white blood cells [17]. OTA-exposed mice have fewer splenic T lymphocytes and mature CD4^+^ cells with more immature double-positive CD4^+^/CD8^+^ cells [18]. OTA exposure also decreases IL-2 production and IL-2 receptor expression in porcine and human T lymphocyte populations and subpopulations [19,20]. OTA and citrinin as the nephrotoxic mycotoxins are the etiological factor of the fatal human kidney disease and show synergistic lethal responses and suppression of lymphocyte proliferation in a synergistic way [21,22]. In addition to lymphocytes, embryonic exposure to AFB_1_ impairs the functions of phagocytes such as macrophages and neutrophils, via the depression of phagocytic potential, inhibition of antiviral activity, and reduction in chemotactic responses [23,24,25]. AFB_1_ also interferes with the innate immunity of macrophages by suppressing tumor necrosis factor-α (TNF- α), interleukin (IL)-1, and IL-6, resulting in the disruption of pulmonary and systemic host defenses [11,26]. Exposure to fumonisin B_1_ (FB_1_) also alters the morphology and functions of macrophages in chickens, leading to increased susceptibility to bacterial infections [27]. Additionally, natural killer cell-mediated cytolysis and resistance to infectious agents are also reduced by exposure to aflatoxins [28,29,30,31] and deoxynivalenol (DON) [32].

Humoral immune responses are also affected by mycotoxin exposure. FB_1_-producing *Fusarium moniliforme* can lead to deficiencies in antibody titers in the chicken immune system [33]. Moreover, the white blood cell population and antibody production are reduced in DON-exposed mice compared with unexposed mice [34,35]. In particular, IgM and delayed-type hypersensitivity responses to infectious bacteria are significantly suppressed. However, serum IgA levels increase after exposure to DON, leading to mesangial deposition of the IgA-immune complex, although serum IgM levels decrease [34]. Other animals, such as chicks and pigs, exhibit increased antibody responses after exposure to DON [36,37]. Depending on the dose regime, the immune response can be differentially regulated by mycotoxins. Low-dose exposure to DON or other type B trichothecene mycotoxins, including nivalenol, 15-acetyl DON, and 3-acetyl DON, induces chemokine production in human or mouse intestinal epithelial cells (IECs) [38,39], IL-2 production in human lymphocytes, and pro-inflammatory cytokine production, including IL-8, IL-6, and TNF-α in human macrophages [40]. Therefore, human and animal immune responses are altered under exposure to mycotoxins, which leads to impaired pathological damages to mycotoxin-exposed tissues or organs.

## 3. Interaction between Mycotoxins and Pathogenic Infections in the GI Tract

### 3.1. Relationship between Mycotoxin Exposure and Salmonella Infection

*Salmonella* is a genus of flagellated, rod-shaped bacteria of the *Enterobacteriaceae* family. The genus consists of three major species, *Salmonella enterica* (*S. enterica*), *S. bongori*, and *S. subterranean*. *Salmonella* in the gastrointestinal (GI) tract are pathogenic bacteria that trigger diarrhea, fever, vomiting, and abdominal cramps, and are sometimes related to postinfectious irritable bowel syndrome [41]. In addition, Salmonellosis is a risk factor of inflammatory bowel disease (IBD) and *Clostridium difficile* infection, which damages intestinal mucosal tissues [42,43]. *S. enterica* serotype Typhimurium (*S.* Typhimurium) triggers pro-inflammatory IL-8 expression and production via MAPK (in particular, p38) activation using the type III secretion system in IECs. The intake of a low concentration of DON renders IECs more susceptible to infection by *S.* Typhimurium and subsequent mucosal inflammatory responses owing to increased *S.* Typhimurium translocation [44,45,46]. Co-exposure to DON (≤0.5 μg/mL, *in vitro*) and *Salmonella* superinduces the expression of intestinal pro-inflammatory cytokines in porcine ileal tissues, resulting in detrimental inflammatory insults in humans and other animals [6]. Pigs exposed to a high dose of the type A trichothecene T-2 toxin via feed have decreased colonization of *S.* Typhimurium in the jejunum, ileum, and colon. However, a low concentration (1–100 ng/mL, *in vitro*) of T-2 toxin enhances the susceptibility of porcine macrophages and IECs to *S.* Typhimurium invasion [47]. Moreover, T-2 toxin-exposed pigs have increased translocation of *S.* Typhimurium through IEC monolayers. Although T-2 toxin favors infection by *S.* Typhimurium, it has adverse effects on the motility and metabolic activity of *S.* Typhimurium, suggesting both deleterious and favorable interactions between T-2 toxin and *S.* Typhimurium [48,49]. T-2 toxin has a profound negative effect on the ability of chickens to resist salmonellosis, but this is not accompanied by marked alterations in T- or B-cell responses to mitogenic stimulation [50]. In mice, increased mortality in response to *S.* Typhimurium challenge is dependent on T-2 toxin (≤1 mg/kg, *in vivo*) in a dose-dependent manner [51]. The increased mortality is mediated by increased splenic counts in mice challenged with *S.* Typhimurium, and T-2 toxin (1 mg/kg, *in vivo*) accelerates body-weight loss in infected mice [51]. In addition, challenge with T-2 toxin (1 mg/kg, *in vivo*) increases *S.* Typhimurium -related lesions in the spleens, kidneys, and livers, but Peyer’s patches and ileal tissues are marginally affected [52]. A high dose of OTA (3 mg/kg, *in vivo*) also increases intestinal colonization of *S.* Typhimurium in young chickens [53,54] and FB_1_ (150 mg/kg, *in vivo*) exacerbates clinical symptoms, such as diarrhea with bloody discharges, induced by *S. enterica* serotype Gallinarum (*S.* Gallinarum) infection [55]. Additionally, quail mortality is increased by FB_1_ (150 mg/kg, *in vivo*) from *Fusarium moniliforme* [55].

Macrophages play a crucial role in the pathogenesis of *Salmonella* infections because the bacteria are able to survive and multiply intracellularly after cellular entry. Macrophage invasion coincides with membrane ruffles, bacterium uptake, and the formation of *Salmonella-*containing vacuoles [56,57]. Although DON (0.25 μg/mL, *in vitro*) does not influence growth or the expression of *S.* Typhimurium virulence genes in macrophages, it promotes invasion and intracellular survival of *S.* Typhimurium in macrophages. Mechanistically, enhanced uptake of *S.* Typhimurium into macrophages by DON coincides with F-actin reorganization of cells. This is mediated by extracellular signal-regulated protein kinase 1/2 (ERK1/2), resulting in increased susceptibility of pigs to infection with *S.* Typhimurium [5]. Although peritoneal macrophages in mice exposed to T-2 toxin (0.1 μM, *in vitro*) do not influence lethality, lethality is significantly higher in *S.* Typhimurium-challenged mice exposed to T-2 toxin (2 mg/mL, *in vitro*) than in mice that are not exposed to T-2 toxin, suggesting that additional immune-related cells are involved in the pathogenesis of *S.* Typhimurium-induced lethality of hosts that are pre-exposed to T-2 toxin [58]. In most infection models, mycotoxins enhance *Salmonella* infections in macrophages, and increase inflammatory responses induced by *Salmonella* infections via the upregulation of pro-inflammatory cytokines and chemokines. In addition, *Salmonella*-induced inflammatory responses are enhanced by mycotoxins, and enhanced inflammatory responses may be a risk factor of inflammatory diseases, such as IBD. As a result of increased colonization, mycotoxins may promote *Salmonella*-induced pathogenicity, e.g., inflammatory responses, by synergistically inducing excessive pro-inflammatory cytokines or chemokine storms.

### 3.2. Relationship between Mycotoxin Exposure and Escherichia Coli Infection

*Escherichia coli* is a gram-negative, rod-shaped bacterium that is found in the lower intestine of mammals. Enterovirulent *E. coli* can be classified based on virulence and acquired genetic features. Enterotoxigenic *E. coli* (ETEC) produce one or more enterotoxins that are heat labile (LT-1 and LT-2) and secrete heat stable enterotoxins (STa and STb). Enteropathogenic *E. coli* (EPEC) harbor a pathogenicity island that encodes a series of proteins involved in the effacement lesion of the intestinal villi of cells. Enteroinvasive *E. coli* (EIEC) have biochemical, physiological, and generic properties that are similar to those of *Sshigella*, invading the epithelial cells of the colon. Enterohemorrhagic *E. coli* (EHEC) secrete Shiga-like toxins and cause severe diseases, such as bloody diarrhea and hemolytic uremic syndrome in humans [59,60,61,62,63,64,65,66,67,68,69,70]. A low level of aflatoxin (1–3 ppb, *in vivo*) or fumonisin (50–350 ppb, *in vivo*) increases cytotoxicity of Shiga-like toxin-producing *E. coli* in calves [71]. In pigs, dietary FB_1_ (0.5 mg/kg, *in vivo*) significantly increases colonization of EPEC strains in the small and large intestines, and subsequent bacterial translocation to extraintestinal organs, including the mesenteric lymph nodes, lung, liver, and spleen [72]. FB_1_ (1 mg/kg, *in vivo*) also downregulates antigen-specific immune responses induced by ETEC infections, resulting in longer shedding of ETEC following infection [73]. Combined exposure to FB_1_ (≤200 mg/kg, *in vivo*) and moniliformin (≤100 mg/kg, *in vivo*) (which is a mycotoxin produced by *Fusarium* species that regulates plant growth and is phytotoxic in maize and tobacco) reduces pathogenic *E. coli* clearance in poultry [74]. In broiler chickens, OTA (80 mg/kg, *in vivo*) worsens *E. coli* infection-mediated diseases, such as perihepatitis and pericarditis, and the fibrin layer becomes thicker after *E. coli* infection [75]. OTA feeding leads to severe swelling of the proximal convoluted tubules and degeneration of the tubular epithelium in *E.coli*-inoculated kidney. In this co-exposure model, degenerative and mononuclear cell infiltration are also observed in the liver [75]. Taken together, exposure to some mycotoxins interferes with host defenses against enterovirulent *E. coli*, leading to the failure of bacterial clearance and promoting mucosal colonization and invasion as well as inflammatory responses. Therefore, mycotoxin exposure can make hosts more susceptible to *E. coli* infection-mediated acute and chronic diseases, including hemolytic uremic syndrome and renal failure.

### 3.3. Relationship between Mycotoxin Exposure and Clostridium Perfringens Infection

*Clostridium perfringens* (*C. perfringens*) is a gram-positive, rod-shaped, spore-forming bacterium and is a risk factor for necrotic enteritis in broiler chickens [76,77,78,79]. *C. perfringens* has five toxinogenic types (A, B, C, D, and E) that are differentiated according to the production of four different toxins (Alpha, Beta, Epsilon, and Iota) [80]. Necrotic enteritis is one of the most important enteric diseases in poultry and is caused by type A *C. perfringens* isolates and rarely by type C isolates [81,82]. Type C toxins of *C. perfringens* are risk factors for several enteric diseases, such as hemorrhagic or necrotic enterotoxemia in piglets, lambs, and calves, and increase vaccination costs in the agricultural industry [81,83]. DON exposure (3000–4000 μg/kg, *in vivo*) at concentrations below the European maximum guidance level of 5000 μg/kg feed in poultry via food intake is a predisposing factor for severe intestinal barrier disruption and enhanced growth and toxin production of *C. perfringens*, resulting in the development of necrotic enteritis in broiler chickens [84]. Necrotic enteritis lesions are mainly distributed in the duodenum and jejunum; these are also the major absorption sites for DON, which affects the functions of the proximal part of the intestinal tract by reducing villus height in the duodenum. This is associated with impaired nutrient uptake or digestion via a reduction in differentiated IECs [84]. In addition, FB_1_ treatment (25 μg/mL, *in vitro*) enhances susceptibility to the toxicity of the epsilon toxin of *C. perfringens*, which mediates cell death in canine cells [85]. Taken together, severe enteric diseases caused by *C. perfringens* may be more likely after exposure to mycotoxins, particularly necrotic enteritis and hemorrhagic or necrotic enterotoxemia. Mechanistically, the increased predisposition to enteric disorders is associated with intestinal barrier disruption and increased cytotoxicity in the gut epithelium in response to mycotoxins.

### 3.4. Relationship between Mycotoxins and Reovirus Infection

Reoviruses (respiratory enteric orphan viruses) are nonenveloped dsRNA-containing viruses that are ubiquitous and infect many mammalian species, including humans and mice [86,87,88,89]. In humans, reoviruses are not associated with any diseases that are more severe than a mild enteric or respiratory illness; however, in mice and rats, they cause many disease syndromes affecting major vital organs, like the brain and heart [90,91]. Enteric reovirus infections produce a self-limited infection in which the virus is cleared from the GI tract within 7 to 14 days after onset [92,93]. Mucosal responses to enteric reovirus infections involve the induction of intestinal IgA and serum IgG as well as cellular immune responses [94,95,96,97,98]. The titers of infected reovirus in the GI tract are 10-fold higher in DON-exposed mice than in control mice after 5 days. In addition, DON (10 and 25 mg/kg, *in vivo*) increases the severity of the reovirus infection and shedding in feces as well as reovirus IgA responses. Elevated shifting to IgA occurs due to the suppression of Th1 and enhancement of Th2 cytokine expression in the presence of DON (10 and 25 mg/kg, *in vivo*). In addition, DON exposure (10 and 25 mg/kg, *in vivo*) makes viral clearance difficult in reovirus-infected hosts [99]. A high concentration of T-2 toxin (1.75 mg/kg, *in vivo*) also results in the suppression of reovirus-induced immune responses, and is associated with reduced clearance of the infected virus [100]. Thus, DON and T-2 toxin promote intestinal viral accumulation and the subsequent elevation of inflammatory insults to the host [100]. Trichothecene mycotoxins diminish cell-mediated viral clearance by enhancing IL-4, IL-6, and IL-10, despite the suppression of interferon-γ (IFN-γ) from Peyer’s patches [99,100]. Taken together, trichothecene mycotoxins are a predisposing factor for reovirus infections in the intestine owing to increases in innate and adaptive immunity, as evidenced by elevated pro-inflammatory cytokines, intestinal IgA, and serum IgG.

## 4. Interaction between Mycotoxins and Pathogen Infections in Respiratory Organs

Viral illnesses are the most common risk factors for upper respiratory symptoms, which affect the nasal cavity, paranasal sinuses, pharynx, and larynx [101]. In contrast, bacterial infections may develop after viral infections and affect upper and lower respiratory systems [101]. *Bordetella bronchiseptica* (*B. bronchiseptica*) and *Pasteurella multocida* (*P. multocida*) are highly prevalent airway pathogenic bacteria and contribute to multiple pathologies in respiratory diseases, such as pneumonia, which is an inflammatory condition of the lung that affects microscopic air sacs known as alveoli [102,103,104]. *B. bronchiseptica* infection prior to challenge with *P. multocida* results in colonization of the upper respiratory tract and tonsils. *B. bronchiseptica*-infected pigs exposed to FB_1_ (10 mg/kg, *in vivo*) show clinical signs including mild serous nasal discharge, sneezing, panting, and hoarseness, unlike pigs not exposed to FB_1_ [105]. *P. multocida* also increases the risk of pneumonia and the severity of the pathological changes [105]. Neither FB_1_ exposure (0.5 mg/kg, *in vivo*) nor infection with *P. multocida* affects weight gain or causes serious clinical signs or lung lesions, and both have minimal effects on the production of bronchoalveolar lavage fluid in pigs. However, co-treatment with FB_1_ and *P. multocida* leads to delayed growth, induced cough, increased bronchoalveolar lavage fluid-producing cells (such as macrophages and lymphocytes), and prominent lung lesions with increased levels of proinflammatory cytokines, such as TNF-α, IFN-*γ*, and IL-18 [106]. Another bacterium, *Mycoplasma hyopneumoniae* (*M. hyopneumoniae*), is a primary pathogen of enzootic pneumonia and causes huge economic losses in the pig industry [107]. FB_1_-fed (20 ppm, *in vivo*) pigs show enhanced infection with *M. hyopneumoniae*, which causes pulmonary inflammatory responses and subsequent severe illness that requires euthanasia and progressive pathology in pigs [108]. As another infective agent affected by fumonisin exposure, porcine reproductive and respiratory syndrome virus (PRRSV) is a pathogenic virus in pigs; it has an enveloped, positive-sense, and single-stranded RNA genome [109]. PRRSV is classified into genotypes 1 and 2 based on 3′-terminus structural genes or the entire genome [110,111]. Both genotypes cause reproductive failure, respiratory signs, and immunosuppression in pigs, and increase mortality of neonatal pigs [112]. PRRSV is an infectious virus that replicates within macrophages or monocytes with the lung. FB_1_ exposure (≤20 ppm, *in vivo*) aggravates the severe symptoms induced by PRRSV in a dose-dependent manner [113]. In conclusion, exposure of pigs to FB_1_ can increase the inflammatory responses to infection via the airway pathogens, affecting animal health and growth. Therefore, further studies are needed to investigate how these interactions between mycotoxins and pathogen infections affect host defense and disease progress to maximize agricultural production.

## 5. Effects of Mycotoxin-Pathogen Exposure on Chronic Mucosal Disorders

Chronic exposure to environmental mycotoxins exacerbates infectious pathogen-induced diseases. Some trichothecene mycotoxins, such as DON and T-2 toxin, can directly damage mucosal tissues via disruption of the gut epithelial barrier and subsequently facilitate the translocation of gut commensal microbiota, pathobionts, and pathogens. In particular, disrupted junctional proteins and epithelial cell death owing to mycotoxins may account for impaired epithelial barrier integrity, which in turn enhances bacterial or viral pathogen infections and subsequent inflammatory insults [114,115,116,117]. Moreover, mycotoxin exposure can be an etiological factor of environmental IBD and has similar mechanistic patterns, including disruption of the intestinal epithelial barrier due to the reduction and delocalization of junctional proteins, and facilitated luminal or external bacterial translocation, triggering excessive immune cell-related intestinal inflammation [44,45]. Airway exposure to mycotoxins may trigger pro-inflammatory responses observed in pneumonia and may facilitate infection of pneumonia-triggering pathogens, including *B. bronchiseptica* and *P. multocida.* In particular, *P. multocida* induces systemic infections and mediates cirrhosis, cellulitis, and increases risk level in patients with organ transplants. Moreover, chronic obstructive pulmonary disease found in the mucosa of larger bronchi is one of the most prevalent diseases in the world, and is exacerbated by bacterial infection [49]. Mycotoxin exposure is another etiological factor in the exacerbation of chronic obstructive pulmonary disease; it increases microbial infection, colonization, or excessive cytokine production via cytoskeletal reorganization mediated by various factors including myosin light chain kinase (MLCK) leading to pulmonary epithelial disruption, one of major pathological events in patients with chronic obstructive pulmonary disease (COPD) [118]. Although extensive studies have examined the association between mycotoxin exposure and infectious diseases, the specificity and chronicity of the related mucosal disorders still requires extensive observations with a broad spectrum of dose regimes and exposure durations in optimized animal models and animal-alternate systems.

## 6. Summary and Final Remarks on the Regulatory Implication

Mucosal exposure to mycotoxins in the GI and respiratory tracts has substantial effects on susceptibility to infectious agents. However, each mycotoxin leads to different biological events in response to mucosal infections, depending on the dose regime and host specificity (Table 1). (1) DON and T-2 toxin enhance susceptibility to *S.* Typhimurium infection by promoting its translocation and intercellular survival via F-actin reorganization in macrophages. In broiler chickens, DON enhances *C. perfringens* growth and toxin production. Reovirus-mediated immune responses are also regulated by exposure to DON in mice, including increased IgA responses, suppressed viral clearance, and Th2 cytokine polarization. (2) FB_1_ aggravates *S.* Gallinarum-induced bloody diarrhea and mortality in quails. In addition, FB_1_ reduces antigen-specific immune responses to pathogenic *E. coli* infections and its clearance. Moreover, FB_1_ exacerbates the severity of damages caused by respiratory tract-associated pathogens in pigs. (3) OTA also increases the colonization of *S.* Typhimurium in chickens, and aflatoxin increases EPEC/EHEC-mediated intoxication by enterotoxins and pore-forming toxins in calves.

Ultimately, studies on the interaction between mycotoxin and pathogens implicate the need for appropriate regulatory limits of mycotoxins in the food and feed for the human and animal population with underlying infectious diseases. Although the various levels of mycotoxin regulatory limits are already set up (Table 2), these are based on the toxicological risk assessment in a healthy population. People or animals with infectious diseases can be more resistant or susceptible to mycotoxicosis than the healthy population. Moreover, the mycotoxin-pathogen interaction in a population with non-pathogenic underlying diseases, including metabolic, cardiovascular, and oncological diseases, needs to be addressed in future investigations.

**Table 1:** Interactions between mycotoxins and mucosal pathogens.

Mycotoxins	Pathogens	Effects	Host
DON	*S.* Typhimurium	Increase in translocation [44,45,46]	Pig
		Increase in cytokine production [6]	
		Promotion of invasion and intracellular survival [47]	Porcine
		Increased susceptibility to infection by F-actin reorganization in macrophages [5]	
T-2 toxin	*S.* Typhimurium	Enhancement of infection [6]	Pig
		Reduction of bacteria mortality [48,49]	
	*S.* Typhimurium	Increase in mortality [51]	Mouse
		Loss of body weight [51]	
		Increased lesions in spleen, kidney and liver [52]	
	*S.* Typhimurium	Increase in colonization [53]	Chicken
OTA	*S.* Typhimurium	Increase in colonization [53,54]	Chicken
FB_1_	*S.* Gallinarum	Diarrhea with bloody discharges and increase in mortality [55]	Japanese quail
FB_1_	EPEC/EHEC	Increase enterotoxin and pore-forming toxin activity [71,73]	Calf
	EPEC	Increase in colonization [72]	Pig
		Translocation to mesenteric lymph nodes, lung, liver, and spleen [72]	
	ETEC	Reduction of antigen-specific immune responses via longer shedding of ETEC [73]	Pig
FB_1_ and Moniliformin	*E.coli*	Reduction of bacteria clearance [74]	Poultry
Aflatoxin	EPEC/EHEC	Increase in enterotoxin and pore-forming toxin activity [71]	Calf
OTA	*E.coli*	Swollen proximal convoluted tubules, degradation of tubular epithelium, and intestinal mephritis in kidney [75]	Broiler chicken
		Degenerative and mononuclear cell infiltration in liver [71]	
DON	*C. perfringens*	Enhancement of growth and toxin production [84]	Broiler chicken
FB_1_	*C. perfringens*	Enhancement of susceptibility to epsilon-toxin [85]	Canine
DON	Reovirus	Elevation of IgA responses [99]	Mouse
		Increase in severity of reovirus infection and shedding [99]	
		Suppression of Th1 cytokine expression [99]	
		Enhancement of Th2 cytokine expression [99]	
		Diminished cell-mediated viral clearance [99]	
		Elevation of reovirus-induced cytokine expression [99]	
T-2 toxin	Reovirus	Suppression of reovirus-induced immune responses [99]	Mouse
		Diminished cell-mediated viral clearance by suppression of IFN-γ and elevation of reovirus-induced cytokine expression [99,100]	
FB_1_	*B. bronchiseptica* and *P. multocida*	Increase in extent and severity of pathogenic changes [102,103,104]	Pig
	*B. bronchiseptica*	Clinical changes including mild serous nasal discharge, sneezing, panting, and hoarseness [105]	
	*P. multocida*	Delayed growth, induced cough, and increased BALF cells such as macrophages and lymphocytes [106]	
		Increase in risk of pneumonia and severity of pathological changes [106]	
		Increase in TNF-α, IFN-γ and IL-18 expression [106]	
	*M. hyopneumoniae*	Induction of pulmonary inflammatory responses and enhancement of infection [108]	
	PRRSV	Severe histological lesion of lung [113]	

**Table 2:** Regulatory limits of food and feed mycotoxins in USA and EU.

Foods or Feeds	Nations	Mycotoxin	Products		Limits (μg/kg)		
Food	USA (Action level)	Aflatoxin (B_1_, B_2_, G_1_, G_2_)	Brazil nut, peanut, peanut products, pistachio nut		20		
		Aflatoxin M_1_	Milk		0.5		
	USA (Guidance level)	Fumonisin (B_1_, B_2_, B_3_)	Maize, maize products		2000–4000		
		Deoxynivalenol	Wheat and wheat products		1000		
	EU	Mycotoxins	Cereal and its products		Limits (μg/kg)		
					B1	B_1_ + B_2_ + G_1_ + G_2_	M_1_
		Aflatoxin	Peanut, nut products, grains, and maize		2.0–12.0	4.0–15.0	-
			Raw milk and milk products		-	-	0.05
			Grain products for infants and baby food		0.1	-	-
			Products for infants		-	-	0.025
		Ochratoxin A	Grains, fruits, coffee beans, wine, and grape Juice		2.0–10.0		
			Products for infants		0.5		
		Fumonisin (B_1_+B_2_)	Maize and its products		200–4000		
		Deoxynivalenol	Non-processed grain products		1250–1750		
			Processed grain products		500–750		
			Products for infants		200		
Feed	USA	Fumonisin	Animal feed	Horses and rabbits	1000		
				Swine	10,000		
				Cow, sheep and goat	30,000		
				Ruminants and poultry	15,000		
				Butchering poultry	50,000		
		Deoxynivalenol	Grains and grain products (>50% grain for cow or chicken)		10,000		
			Grains and grain products (>40% grain for cow or chicken)		5000		
			Grains and grain products digested by sheep		5000		
	EU	Aflatoxin B_1_	Complete feeding stuffs for cattle, sheep and goat		20		
			complete feeding stuffs for dairy animals		5		
			complete feeding stuffs for calves and lambs		10		
			Complete feeding stuffs for pigs and poultry (except young animals)		20		
			Other complete feeding stuffs		10		
			Complementary feeding stuffs for cattle, sheep and goats (except complementary feeding stuffs for dairy animals, calves and lambs)		20		
			Complementary feeding stuffs for pigs and poultry (except young animals)		20		
			Other complementary feeding stuffs		5		
		Deoxynivalenol	cereals and cereal products with the exception of maize by-products		8000		
			maize by-products		12,000		
			Complementary and complete feeding stuffs with the exception of maize by-products		5000		
			complementary and complete feeding stuffs for pigs		900		
			complementary and complete feeding stuffs for calves (<4 months), lambs and kids		2000		
		Zearalenone	cereals and cereal products with the exception of maize by-products		2000		
			maize by-products		3000		
			Complementary and complete feeding stuffs				
			complementary and complete feeding stuffs for piglets and gilts (young sows)		100		
			complementary and complete feeding stuffs for sows and fattening pigs		250		
			complementary and complete feeding stuffs for calves, dairy cattle, sheep (including lambs) and goats (including kids)		500		
		Ochratoxin A	cereals and cereal products		250		
			complementary and complete feeding stuffs for pigs		50		
			complementary and complete feeding stuffs for poultry		100		
		Fumonisin B_1_ and B_2_	maize and maize products		60,000		
			complementary and complete feeding stuffs for pigs, horses, rabbits and pet animals		5000		
			complementary and complete feeding stuffs for fish		10,000		
			complementary and complete feeding stuffs for poultry, calves (<4 months) and mink		20,000		
			complementary and complete feeding stuffs for adult ruminants (>4 months) and mink		50		
